# TrIncNet: a lightweight vision transformer network for identification of plant diseases

**DOI:** 10.3389/fpls.2023.1221557

**Published:** 2023-07-27

**Authors:** Pushkar Gole, Punam Bedi, Sudeep Marwaha, Md. Ashraful Haque, Chandan Kumar Deb

**Affiliations:** ^1^ Department of Computer Science, University of Delhi, New Delhi, India; ^2^ Indian Council of Agricultural Research (ICAR)-Indian Agricultural Statistics Research Institute, New Delhi, India

**Keywords:** vision transformer (ViT), inception block, deep learning, automatic plant disease detection, PlantVillage dataset, maize crop

## Abstract

In the agricultural sector, identifying plant diseases at their earliest possible stage of infestation still remains a huge challenge with respect to the maximization of crop production and farmers’ income. In recent years, advanced computer vision techniques like Vision Transformers (ViTs) are being successfully applied to identify plant diseases automatically. However, the MLP module in existing ViTs is computationally expensive as well as inefficient in extracting promising features from diseased images. Therefore, this study proposes a comparatively lightweight and improved vision transformer network, also known as “TrIncNet” for plant disease identification. In the proposed network, we introduced a modified encoder architecture a.k.a. Trans-Inception block in which the MLP block of existing ViT was replaced by a custom inception block. Additionally, each Trans-Inception block is surrounded by a skip connection, making it much more resistant to the vanishing gradient problem. The applicability of the proposed network for identifying plant diseases was assessed using two plant disease image datasets viz: PlantVillage dataset and Maize disease dataset (contains in-field images of Maize diseases). The comparative performance analysis on both datasets reported that the proposed TrIncNet network outperformed the state-of-the-art CNN architectures viz: VGG-19, GoogLeNet, ResNet-50, Xception, InceptionV3, and MobileNet. Moreover, the experimental results also showed that the proposed network had achieved 5.38% and 2.87% higher testing accuracy than the existing ViT network on both datasets, respectively. Therefore, the lightweight nature and improved prediction performance make the proposed network suitable for being integrated with IoT devices to assist the stakeholders in identifying plant diseases at the field level.

## 1 Introduction

The agricultural industry is crucial for overall economic development in India as it contributes approximately twenty percent of the country’s GDP, and roughly 55% of India’s workforce is engaged in agricultural-related activities (Chand and Singh, 2022). As the country’s population is increasing exponentially, the demand for food is also proliferating day by day. The agriculture sector faces many challenges in fulfilling such colossal food demand. Hence, agrarian researchers across the country are actively engaged in developing a sustainable food grain production system. Disease infestation in the crops is one of such challenges as it hampers overall food-grain production and impacts the overall food supply chain. In this context, identifying the plant diseases in their earliest possible stage would be a viable solution that would help in minimizing the crop loss and maximizes the farmer’s income too. Conventionally, farmers and plant pathologists manually examine the plants to detect probable diseases, which is quite a difficult and laborious task. Due to the technological advancements in computer vision, nowadays, plant diseases are being identified with the help of computational techniques and digital visual images of plant leaves.

In order to diagnose plant diseases *via* their symptomatic leaf images, various researchers have applied different Machine Learning (ML) techniques (Khan et al., 2022). Although ML techniques can solve the problem of automatic plant disease recognition *via* digital leaf images, but these methods prominently suffer from two issues. First, these techniques cannot automatically extract various temporal and spatial features of images, which are used in image classification. Second, they are unable to process large image datasets quickly as they are not implemented in such a manner that they can take the computational advantages of Graphic Processing Units (GPUs). In order to conquer the shortcomings of these techniques, researchers have used Deep learning (DL) methods, particularly CNNs, to recognize plant diseases in an automated manner. The CNN automatically extracts various temporal and spatial features from the given image *via* small convolutional filters to classify the images into their corresponding classes. Moreover, it can also take the computational advantage of the GPUs to perform various mathematical operations faster. Therefore, many researchers have used CNN models (either state-of-the-art CNN architectures or customized CNN architectures) to diagnose plant diseases automatically (Atila et al., 2021; Dhaka et al., 2021; Tiwari et al., 2021).

Vaswani et al. (2017) proposed the Transformer model that revolutionized the natural language processing domain. Thereafter, Dosovitskiy et al. (2021) designed a novel ViT model for image classification based on the Transformer model. It encompasses multiple encoder blocks connected by a direct connection only. Each encoder block of ViT model is comprised of Multi-Head Attention, Layer Normalization, and MLP modules. Dosovitskiy et al. (2021) found that it outperformed various state-of-the-art CNN architectures. Therefore, researchers have applied the ViT model to detect plant diseases automatically (Borhani et al., 2022). Despite of great performance of the ViT model, this model suffers from a major drawback that it contains an MLP module in its encoder block which is computationally expensive as well as inefficient in extracting various temporal and spatial features from the images under study. Therefore, we addressed these drawbacks and developed a comparatively less computationally expensive ViT network for diagnosis of plant diseases. The major contribution of the present study has been provided below:

• We proposed a computationally lightweight and improved Vision Transformer (ViT) network also known as “TrIncNet” for image-based plant disease detection. The TrIncNet model is composed of multiple modified encoder blocks aka Trans-Inception blocks. Each Trans-Inception block comprises of inception module in place of MLP module for extracting various temporal and spatial features from leaf images. Additionally, skip connections are added between each Trans-Inception block to make the proposed network more resistant towards the vanishing gradient problem.
• The proposed network has been trained and tested on two plant disease image datasets viz: PlantVillage dataset (Mohanty et al., 2016) and in-field Maize image dataset (Haque et al., 2022) for showcasing their applicability in the real-world scenario. Moreover, we performed the comparative performance analysis of the proposed network with the existing state of the art networks (e.g., ViT, VGG-19, GoogLeNet, ResNet-50, Xception, InceptionV3, MobileNet, etc) on both the dataset.

This paper is organized into eight sections. Section 1 (current section) highlights the devastating impact of plant diseases, importance of crop protection, constraints of the conventional approaches of disease detection and management, importance of computer vision based technologies *etc.*: Section 2 explores and discusses the relevant works related to the current study, Section 3 explains and describes the approach of proposed model development; Section 4 describes the dataset and experimental setup used in the present study; Section 5 presents the experimental results and finding of the current study; Section 6 briefly discusses and interprets the results of the current studies; and section 7 concludes the whole study and aligns the future perspective of this study.

## 2 Related works

Many research efforts are made in the literature to automatically identify plant diseases *via* their digital leaf images. Earlier, researchers have applied different ML techniques (Trivedi et al., 2020; Varshney et al., 2021). Though ML methods effectively identify plant diseases, but they suffer from two main limitations. First, they are unable to capture the various spatial and temporal features of images automatically. Second, processing large image datasets can be slow and time-consuming with traditional ML techniques as they are not developed in a way that they can leverage the advantages of GPUs. To conquer the shortcomings of ML algorithms, researchers have utilized DL methods, particularly CNNs, to identify plant diseases automatically. For example, Mohanty et al. (2016) analyzed the performances of GoogLeNet and AlexNet architectures and found that GoogLeNet outperformed AlexNet with 99.34% testing accuracy. Other research works (Sakkarvarthi et al., 2022; Biswas and Yadav, 2023) used different state-of-the-art CNN architectures to identify plant diseases. Haque et al. (2021) used GoogLeNet architecture to recognize the Maydis Leaf Blight (MLB) disease in Maize crops. They used real-field Maize leaf images for model training and got 99.14% testing accuracy. In another research work, Haque et al. (2022) investigated the effect of the dense layer, global average pooling layer, and flatten layer on the performance of InceptionV3 (Szegedy et al., 2016) model in detecting three types of diseases in Maize plants. Nigam et al. (2023) experimented with eight EfficientNet-based CNN architectures to identify Stem Rust, Stripe Rust, and Leaf Rust diseases in wheat plants. They found that EfficientNet-B4 CNN architecture outperformed other architectures with 99.35% testing accuracy.

Some researchers tried to build a lightweight DL model for the plant disease diagnosis. Bedi and Gole (2021a) developed a hybrid model with the help of Convolutional Autoencoder (CAE) and CNN to identify peach plants’ Bacterial Spot disease, and their model attained 98.38% testing accuracy. In (Bedi and Gole, 2021b), an effort was made by authors to increase the accuracy of peach plants’ Bacterial Spot disease identification to 99.51% by developing a novel DL model by combining the Ghost (Han et al., 2020) and Squeeze-and-Excitation (Hu et al., 2018) modules. Xiang et al. (2021) developed a lightweight network to identify the severity of plant diseases. They designed a lightweight CNN model with the help of multiple-size convolutional filters and channel shuffle operation. Their best model achieved 90.6% accuracy and 84.3% f1-score. Haque et al. (2022) proposed a lightweight custom CNN model for detecting the diseases of maize crop based on the maize dataset from plant data repository. Their proposed network worked quite well on test dataset and obtained 99.1% classification accuracy. Sharma et al. (2023) designed a lightweight DLMC-Net model by using novel collective blocks and passage layers. Moreover, they used depth-wise separable convolution operation to reduce the number of weight parameters. Their proposed DLMC-Net model achieved 93.56%, 92.34%, 99.50%, and 96.56% accuracy in detecting diseases from the leaf images of citrus, cucumber, grapes, and tomato plants, respectively.

In some recent studies, attention-mechanism is also utilized to enhance the efficacy of different DL frameworks. Karthik et al. (2020) applied attention to the ResNet architecture for disease diagnosis in tomato plants and achieved 98% accuracy in detecting ten tomato plant diseases. Chen et al. (2021) embedded channel and spatial attention modules in the DenseNet CNN architecture and used the Depthwise separable convolution operation in place of standard convolution operation. They tested the applicability of their approach in identifying diseases in Maize plants on their own collected dataset and PlantVillage dataset. They reported in the paper that their model attained 95.86% and 98.5% accuracy on their collected and PlantVillage datasets, respectively. Zhao et al. (2022) designed RIC-NET model using Residual and Inception blocks. They used Convolutional Block Attention Module (CBAM) to enhance the RIC-NET model’s performance. Their model identified the diseases in potato, corn, and tomato plants with 99.55% accuracy, as claimed by the authors. Li et al. (2023) designed a novel Muti-Dilated-CBAM-DenseNet (MDCDenseNet) architecture to identify Maize plant diseases in the farmlands. Their proposed model attained 98.84% testing accuracy on the Maize plant leaf images collected from the agricultural fields of Northeastern Agricultural University, China. Naik et al. (2022) used the squeeze-and-excitation network-based CNN model (SECNN) to detect five diseases (down curl of a leaf, Geminivirus, Cercospora leaf spot, yellow leaf disease, and up curl) in Chili plant’s leaf images. Their proposed model attained 98.63% and 99.12% accuracy without augmentation and with augmentation, respectively. Moreover, they tested the model’s performance on the PlantVillage dataset and found that the SECNN model achieved 99.28% accuracy. Pandey and Jain (2022) proposed a novel attention-based learning paradigm to improve the CNN model’s performance in diagnosing plant diseases from leaf images. Their proposed model achieved 99.93% accuracy on the PlantVillage dataset. Kaya and Gürsoy (2023) used the Muti-Head Attention operation in the DenseNet-121 CNN architecture to identify plant diseases and achieved 98.17% accuracy on the PlantVillage dataset.

Due to the powerful capabilities of the Vision Transformer (ViT) model in image classification, Thai et al. (2021) applied the ViT model to identify diseases in the cassava field. They observed that the ViT model outperformed other standard CNN architectures like EfficientNet and ResNet-50 by giving 1% higher accuracy. Another work that utilized the ViT model for plant disease detection was done by Wu et al. (2021). They used the ViT model and a novel multi-granularity feature extraction module to identify tomato plant diseases. As per their paper, the proposed approach outperformed others by roughly 2% higher accuracy. In research work done by Lu et al. (2022), a novel ghost-convolutional Transformer model was proposed to detect diseases in grape plants and attained 98.14% accuracy in identifying eleven grape plant diseases. Some recent studies have combined the ViT and CNN models to solve various computer vision problems. Si et al. (2022) designed Inception-Transformer model for image classification and segmentation tasks. They tested their model on the ImageNet and COCO datasets and found that it surpassed other DL models. Similarly, Bana et al. (2022) designed a Generative Adversarial Network (GAN), which utilized the ViT model and Inception module for image colorization. Another research work done by Zhang et al. (2022) combined the goodness of ViT and CNN models to design a novel Tranvolution model to diagnose plant diseases automatically. They tested their model on the PlantDoc dataset and found that the Tranvolution model outperformed other research works present in the literature.

## 3 Model development

### 3.1 Existing vision transformer network

**Figure 1:**
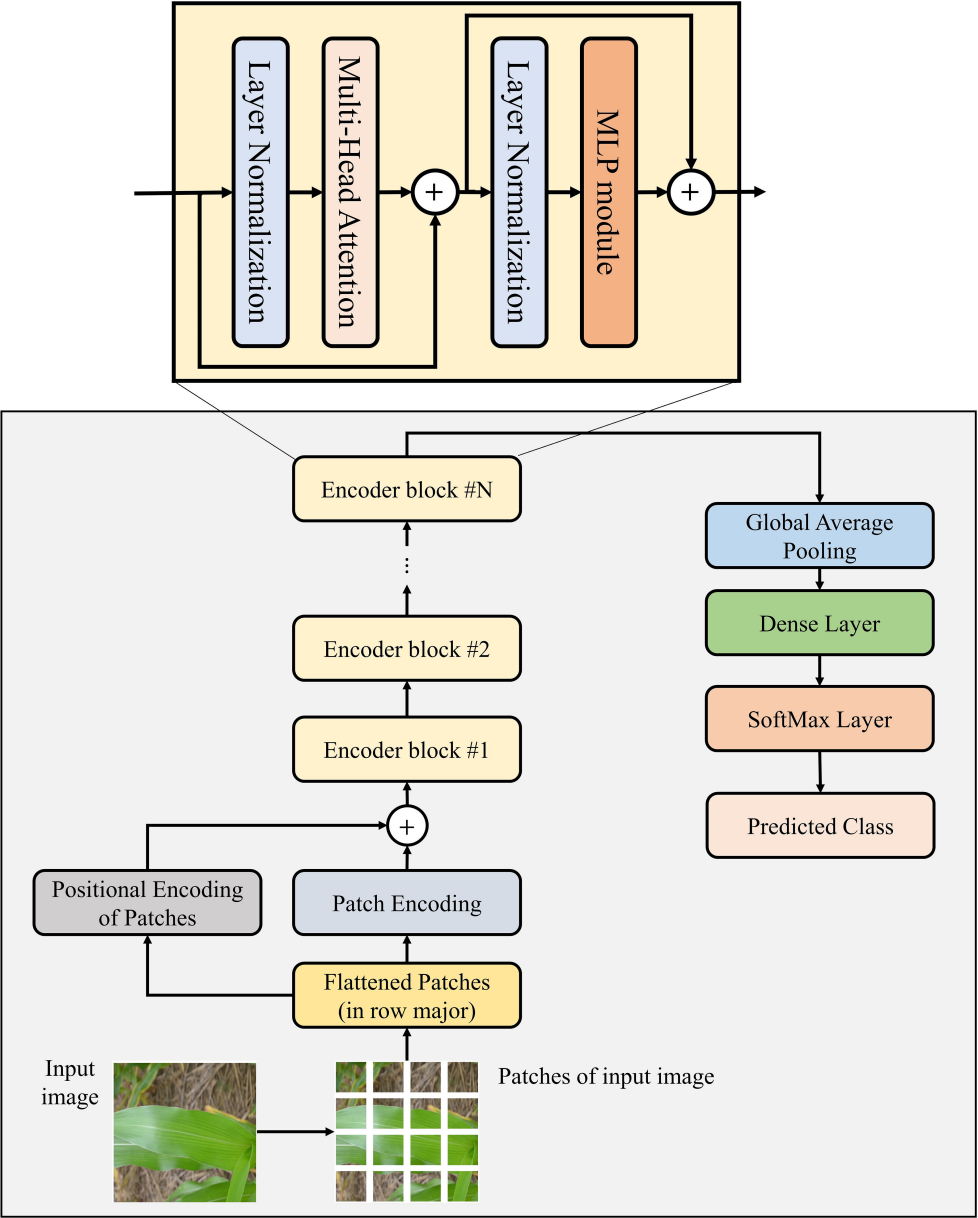
Architectural design of the existing ViT network.

The ViT network is a Transformer (Vaswani et al., 2017) based DL model designed by Dosovitskiy et al. (2021) to perform image classification and segmentation tasks. This model comprises multiple stacked encoder blocks, and each encoder block of the ViT model contains three modules: Multi-Head Attention module, Layer Normalization module, and MLP module. The ViT model’s architectural design is shown in **Figure 1**.

The Multi-Head Attention module performs multiple self-attention operations parallelly, through which the model can capture global dependencies between image patches. The Layer-Normalization module normalizes its previous layer’s activations to improve the model’s stability and performance. The MLP module comprises two densely connected layers that extract various features from image patches. However, the MLP module suffers from a major drawback: all layers of this module are densely connected to each other. Therefore, it requires a huge number of weight parameters to be trained, which makes the ViT model computationally heavy. Moreover, the MLP module is unable to capture the temporal and spatial features of images efficiently and effectively, which can later enhance the performance of model in image classification. Hence, in this study, a novel TrIncNet model has been designed and developed, which fixes these drawbacks of the ViT model, and the description of the TrIncNet model has been given in the next subsection.

### 3.2 Proposed vision transformer network

**Figure 2:**
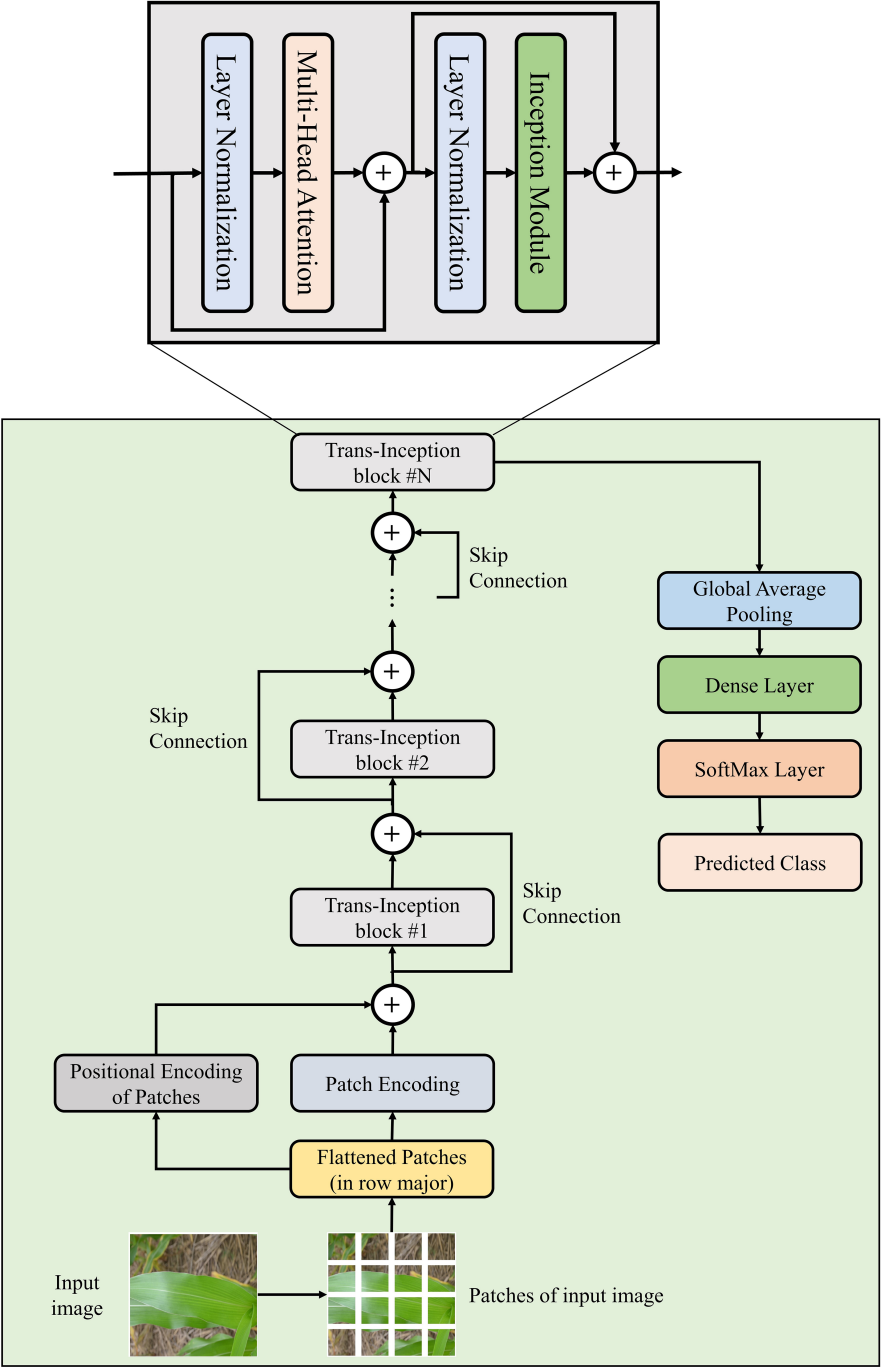
Architectural design of the proposed TrIncNet network.

In order to conquer the shortcoming of ViT model, the MLP module has been replaced with the Inception module in the ViT model’s encoder block, and this modified encoder block is named as “Trans-Inception block”. The reason for using the Inception module in place of the MLP module is that the Inception module performs convolution and max-pooling operations parallelly. Thus, it uses significantly less number trainable weight parameters as compared to the MLP module. Moreover, it can also extract various spatial and temporal features of images effectively and efficiently, which can enhance the performance of the model to perform image classification. In this research work, a novel Trans-Inception Network also known as TrIncNet model is proposed, which comprises of multiple stacked Trans-Inception blocks. As per our best belief, this model has not been proposed in any existing studies present in the literature. Furthermore, each Trans-Inception block of the TrIncNet model is also surrounded by a skip connection which makes the model much more resistant to the vanishing gradient problem. The architectural design of TrIncNet model and its Trans-Inception block has been shown in **Figure 2**.

Each Trans-Inception block of the TrIncNet model comprises three modules: Multi-Head Attention, Layer Normalization, and Inception modules. Out of these three modules, two modules: Multi-Head Attention and Layer Normalization modules are taken from the ViT model’s encoder block, and the Inception module is added to the Trans-Inception block in this research work. All modules of the Trans-Inception block have been described below:

#### 3.2.1 Multi-head attention module

It performs m  self-attention operation parallelly, where m is a hyperparameter representing the number of heads used in this module. In this research work, twelve-headed attention has been used. The phenomenon of human eye perception inspires self-attention operation, as the human eye focuses only on the part of information while ignoring other things. This operation aims to gather the relationship among all patches of an image. Let there be k such patches, i.e., $\left(e_{1},\;e_{2},\;e_{3},\;\ldots ,\;e_{k}\right)$ represented by $E\in {\mathbb{R}}^{k\times d}$ , where d is the embedding dimension in which the information of each patch has been embedded. In self-attention operation, three learnable weight matrices: Query ( $W_{Q}\in {\mathbb{R}}^{d\times d_{q}}$ ), Key ( $W_{K}\in {\mathbb{R}}^{d\times d_{k}}$ ), and Value ( $W_{V}\in {\mathbb{R}}^{d\times d_{v}}$ ) are trained using the backpropagation algorithm, where $d_{q},d_{k},$ and $d_{v}$ are the number of columns present in Query, Key, and Value weight matrices. In self-attention operation, first, the input sequence E is multiplied with these learnable matrices to get $Q=EW_{Q}$ , $K=EW_{K}$ , and $V=EW_{V}$ matrices. After obtaining the Q , K , and V matrices, the self-attention score ( Z ) matrix is calculated by equation 1 (Vaswani et al., 2017).


(1) $$Z=softmax\left(\frac{QK^{t}}{\sqrt{d_{k}}}\right)\cdot V$$

The outputs of all m heads are concatenated together and then multiplied by an output weight matrix ( $W_{O}\in {\mathbb{R}}^{k\times md_{v}\;}$ ) according to equation 2, where $Z_{i}$ is the self-attention score matrix of $i^{th}$ head.


(2) $$Z_{multihead}=concat{\left(Z_{1},Z_{2},\ldots ,Z_{i},\ldots ,\;Z_{m}\right)}^{t}W_{O}$$

#### 3.2.2 Layer normalization module

To conquer Batch Normalization’s shortcomings, Ba et al. (2016) proposed the Layer Normalization technique. This technique normalizes the activations in the feature direction instead of the batch direction. Hence, it removes the shortcoming of Batch Normalization by removing the dependence on batches. Moreover, it normalizes every feature of the activations to unit variance and zero mean. In the Layer Normalization paradigm, first, means and variances are calculated for each channel of the feature map as per equation 3 and equation 4, respectively. Second, the normalized feature maps are computed by equation 5, and at last, scaling and shifting are done with the help of two learnable parameters, i.e., γ and β , by equation 6.


(3) $$\mu_{d,c}=\frac{1}{HW}\sum_{h=1}^{H}\sum_{w=1}^{W}x_{dhwc}$$

(4) $$\sigma_{d,c}^{2}=\;\sum_{h=1}^{H}\sum_{w=1}^{W}{\left(x_{dhwc}-\mu_{d,c}\right)}^{2}$$

(5) $$x_{d,hwc}^{norm}=\;\frac{x_{dhwc}-\mu_{d,c}}{\sqrt{\sigma_{d,c}^{2}+\epsilon }}$$

(6) $$y_{d}=\gamma \;x_{d}^{norm}+\beta \equiv LN_{\gamma ,\beta }\left(x_{d}\right)$$

where $N_{F}$ denotes the number of the feature maps, $1\leq d\leq N_{F}$ , H , W , and C are the height, weight, and channels of the feature map, respectively, and 1≤c≤C .

#### 3.2.3 Inception module

The Inception module performs three convolutional operations with 5×5 , 1×1 , and 3×3 filters and a 3×3 max-pooling operation simultaneously; therefore, it can extract various temporal and spatial features of images parallelly with different receptive fields. As the Inception module performs convolution and max-pooling operations; thus, it has various advantages over the MLP module, which are listed below:

##### 3.2.3.1 Spatial invariance (Shift invariance)

It refers to the property of the convolution operation, which makes it able to recognize the object in the image irrespective of its position. The convolution operation holds this property because the filter size is much lesser as compared to image size.

##### 3.2.3.2 Local translation invariance

Through this property, the Inception module can recognize the rotated or tilted object in the image. The pooling operation of Inception module helps to achieve this property.

##### 3.2.3.3 Parameter sharing

In convolution operation, weight parameters are shared with the help of convolutional filters. The size of these convolutional filters is much lesser than the image size, and only these filters are trained to extract the images’ features. Hence, the total trainable parameters present in the Inception module are much lesser than those in the MLP.

### 3.3 Asymptomatic analysis on weight parameters used by ViT and TrIncNet models

To analyze the efficiency of the novel Trans-Inception block over the original encoder of the ViT model, asymptomatic analysis has been done on the number of weight parameters used by these blocks. As it can be seen from **Figures 1**, **2**, the Trans-Inception block is different from the original encoder block of ViT only in terms of the Inception module’s presence. Therefore, the asymptomatic analysis is done only between the MLP and Inception modules. Let, $I\in {\mathbb{R}}^{M\times N}$ is the input to the MLP module of the ViT, where M is the number of patches in one leaf image, and N is the size of one embedded patch. As mentioned in (Dosovitskiy et al., 2021), the MLP module present in the encoder block of the ViT model contains two fully connected layers having output sizes 2N and N , respectively. Hence the total number of weight parameters used by the MLP module for one patch of leaf image is $O\left(2N\times N\;+\;N\times 2N\right)\Rightarrow O\left(N^{2}\right)$ , asymptomatically. Similarly, for M number patches, total *O*(*MN* ^2^) weight parameters are used by the MLP module. On the other hand, if F is the maximum number of filters used by any convolution operation of the Inception module, then it requires *O*(max(*M* ^2^ *F* ^2^)) weight parameters asymptomatically (calculated in Appendix). The above analysis shows that the proposed Trans-Inception block requires significantly fewer weight parameters to train than the ViT model’s encoder block. Furthermore, lesser weight parameters used by any model imply that it would require less training time and inference time. Hence, the TrIncNet model needs a smaller amount of inference time and training time as compared to the ViT model. Details of experimentation done in this work are described in the next section.

## 4 Experimental material

### 4.1 Dataset description

The experimentation of this research work is carried out on the Maize dataset (which comprises real-field leaf images having complex backgrounds) and the PlantVillage dataset (which acts as a benchmark dataset for plant disease detection problems). These datasets are described below:

#### 4.1.1 Maize dataset

**Figure 3:**
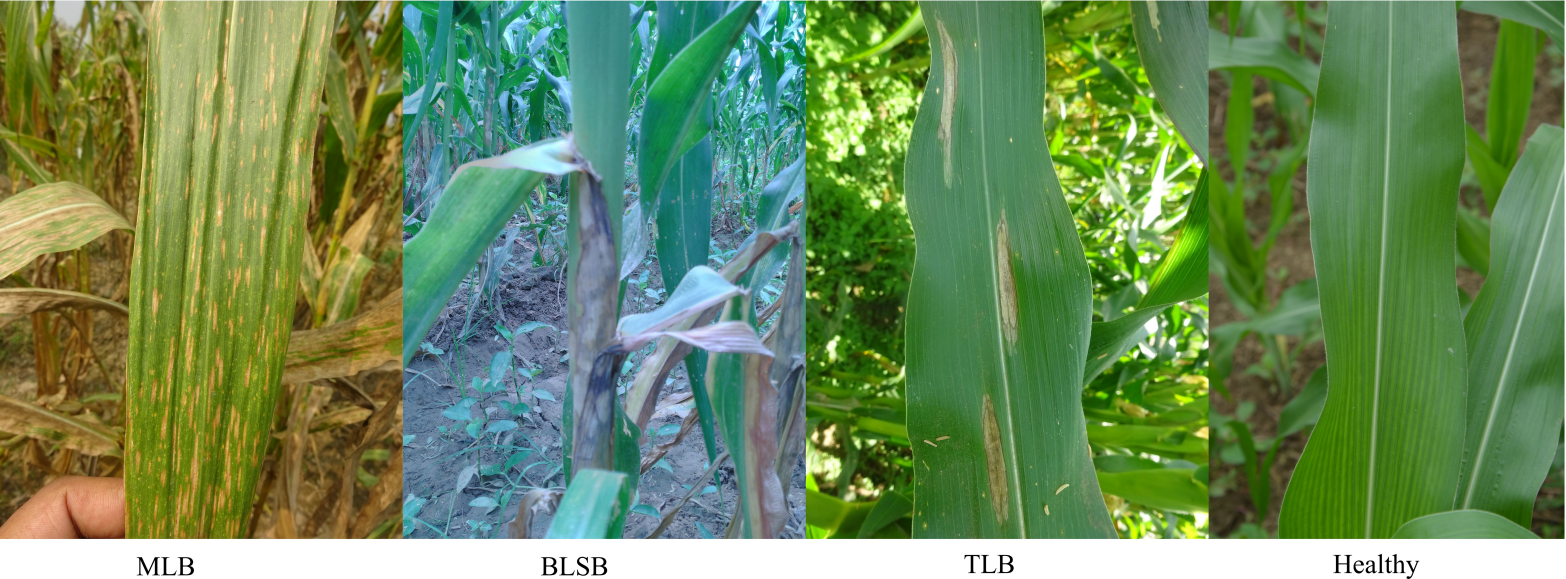
Leaf images from each class of the Maize dataset.

The Maize dataset contains 13,971 leaf images which were captured from multiple agricultural fields of Indian Institute of Maize Research, Ludhiana, India. The images are captured non-invasively by maintaining 25-40 cm distance from the camera device to the affected part of the plant and focused on the top/front view of symptomatic parts of the plant. In this dataset, leaf images of three diseases, i.e., Turcicum Leaf Blight (TLB), Banded Leaf and Sheath Blight (BLSB), and Maydis Leaf Blight (MLB), are present along with the healthy leaf images. Few representative leaf images from each class of the dataset are shown in **Figure 3**.

#### 4.1.2 PlantVillage dataset

**Figure 4:**
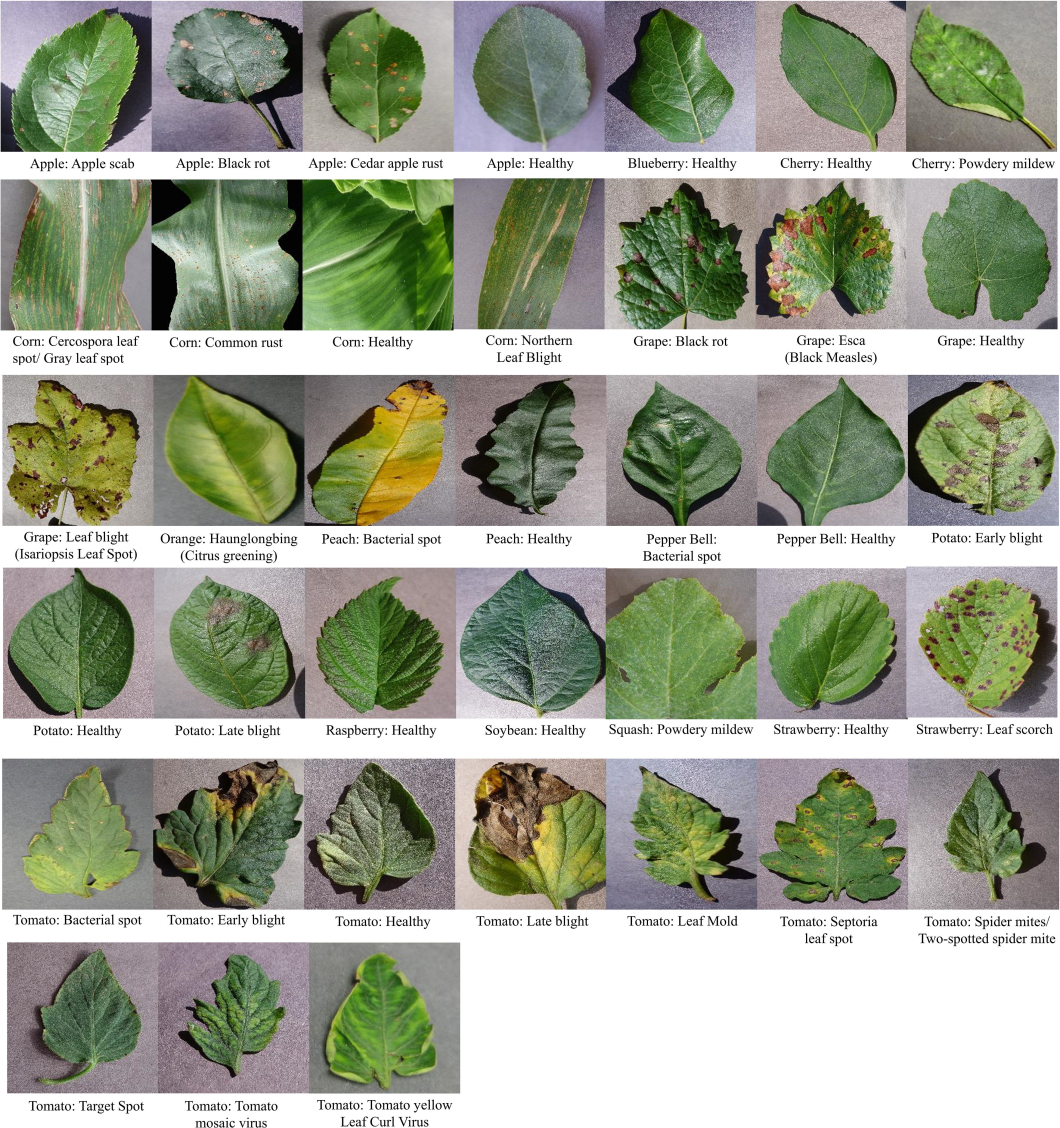
Leaf images from each class of the PlantVillage dataset.

It is a benchmark dataset used to measure the performance of any DL or ML model for automatically recognizing diseases in plants (Mohanty et al., 2016). This dataset contains 54,503 leaf images of 14 plant species which have been categorized into 38 classes. Few representative leaf images from each class of the dataset are shown in **Figure 4**.

### 4.2 Data preprocessing

It is an important step in designing a DL framework for automatically diagnosing diseases in plants. In this research work, following data preprocessing techniques have been used:

#### 4.2.1 Image resizing

It is used to either increase or decrease the image’s resolution. The major advantage of image resizing is that it speeds up the DL model’s training process. Therefore, the images of both datasets have been resized to 256 × 256 dimension *via ImageDataGenerator* class of the Keras library.

#### 4.2.2 Image normalization

The pixel value varies between 0 to 255, and using these pixel values while training the Deep Learning model, computation becomes complex for high pixel values. Therefore, pixel values of images should be normalized between 0 and 1. This can be done by dividing each image pixel value by 255.

#### 4.2.3 Data augmentation

DL models need a large amount of data to generalize a model or prevent the overfitting problem. Data augmentation is a process that increases the dataset’s size by applying various image processing techniques like rotation, flipping, etc. (Bedi et al., 2021). There are two types of data augmentation: online data augmentation and offline data augmentation. In offline data augmentation, the output images are saved on disk after going through the various image processing operations and then used for model training. Whereas in online data augmentation, the transformed images are directly used in model training. Since the leaf images present in the Maize dataset are fewer in number and during model training, it causes model overfitting. Therefore, in order to tackle this problem, the size of the Maize dataset is artificially increased *via* offline data augmentation. After augmentation, the Maize dataset has 100000 leaf images.

#### 4.2.4 Data Splitting

In this step, the leaf images of both datasets are randomly split into the training subset, validation subset, and test subset as per the 70:15:15 ratio. The training subset’s leaf images are utilized for training the models, and validation subset has been utilized to adjust the values of hyperparameters so that the best-performing model can be achieved. Finally, the test subset is utilized for measuring the TrIncNet model’s effectiveness on unseen leaf images.

### 4.3 Experimental setup

The Nvidia DGX Server having Intel(R) Xeon(R) CPU with 528 GB RAM and NVidia Tesla V100-SXM2 32 GB Graphic Card, is used to carry out the experiments of this research work. Python programming language is used to write the scripts for the experiments; however, any programming language can be used for experimentation. The *Keras* Python library embedded in *Tensorflow 2.6.0* has been utilized to develop the TrIncNet model, the ViT model, and six state-of-the-art CNN architectures.

The TrIncNet model’s performance is compared with the ViT (Dosovitskiy et al., 2021) model and six state-of-the-art CNN architectures: VGG-19 (Simonyan and Zisserman, 2015), GoogLeNet (Szegedy et al., 2015), ResNet-50 (He et al., 2016), Xception (Chollet, 2017), InceptionV3 (Szegedy et al., 2016), MobileNet (Howard et al., 2017). These models are trained for 500 epochs and 32 batch size using Adam optimizer to minimize the categorical cross-entropy loss between the logits and actual labels of leaf images. Early stopping with patience value 20 is used to prevent model overfitting., i.e., if validation accuracy is not improved for twenty consecutive iterations, then model training would stop.

As already discussed, the TrIncNet model is designed by replacing the MLP model with the Inception module in the encoder block of the ViT model. Therefore, in order to examine the effect of this replacement on number of weight parameters, ViT and TrIncNet models are implemented using the hyperparameters given in **Tables 1**, **2**, respectively. These values for different hyperparameters of the ViT and TrIncNet models have been derived *via* extensive experimentation. The layer-wise implementation details of ViT and TrIncNet models have been tabulated in **Tables 3**, **4**, respectively.

**Table 1:** Values of hyperparameters for the ViT model’s implementation.

Hyperparameter	Value
Image size	256×256
Patch size p×p	16×16
Size of Embedded Patch ( N )	256
Number of Encoder blocks	2
Number of Heads (m)	12
Activation function	SoftMax (Output Layer)
	ReLu (Hidden Layers)
Layer_normalization_rate (epsilon)	$10^{-6}$

**Table 2:** Values of hyperparameters for the TrIncNet model’s implementation.

Hyperparameter	Value
Image size	256×256
Patch size (p×p)	16×16
Size of Embedded Patch ( N )	256
Number of Trans-Inception blocks	2
Number of Heads (m)	12
Activation function	SoftMax (Output Layer)
	ReLu (Hidden Layers)
Layer_normalization_rate (epsilon)	$10^{-6}$
Number of Filters (used in the Inception module)	96, 16, 64, 128, 32, 32
Padding (used in the Inception module)	Same
Stride (used in the Inception module)	1×1

**Table 3:** ViT model’s implementation details.

Layer No.			Layer Name	Input Shape	Connected to	Output Shape	Parameters
1			Input Layer	256×256×3	–	256×256×3	0
2			Patches	256×256×3	Input Layer	256×768	0
3			Patch Encoder	256×768	Patches	256×256	262400
4			Layer Normalization #1	256×256	Patch Encoder	256×256	512
5			Multi-Head Attention #1	256×256	Layer Normalization #1	256×256	3155200
6			Add #1	256×256 , 256×256	Multi-Head attention #1, Patch Encoder	256×256	0
7			Layer Normalization #2	256×256	Add #1	256×256	512
8	**MLP Module #1**		**Dense #1**	256×256	**Layer Normalization #2**	256×512	131584
			**Dense #2**	256×512	**Dense #1**	256×256	131328
9			Add #2	256×256 , 256×256	Dense #2, Add #1	256×256	0
10			Add #3	256×256 , 256×256	Add #2, Patch Encoder	256×256	0
11			Layer Normalization #3	256×256	Add #3	256×256	512
12			Multi-Head attention #2	256×256	Layer Normalization #3	256×256	3155200
13			Add #4	256×256 , 256×256	Multi-Head attention #2, Add #3	256×256	0
14			Layer Normalization #4	256×256	Add #4	256×256	512
15		**MLP Module #2**	**Dense #3**	256×256	**Layer Normalization #4**	256×512	131584
			**Dense #4**	256×512	**Dense #3**	256×256	131328
16			Add #5	256×256 , 256×256	Dense #4, Add #4	256×256	0
17			GlobalAveragePooling1D #1	256×256	Add #5	256	0
18			Dense #5	256	GlobalAveragePooling1D #1	64	16448
19			Dense #6 (Output Layer)	64	Dense #1	4 (for the Maize dataset), 38 (for the PlantVillage dataset)	262 , 2470
**Total Weight Parameters**						**7117382 (for the Maize dataset)** **7119590 (for the PlantVillage dataset)**	

The value written in bold font highlights the layers of MLP module in this table. These layers are the only difference between existing ViT model and our proposed TrIncNet model.

**Table 4:** TrIncNet model’s implementation details.

Layer No.			Layer Name	Input Shape	Connected to	Output Shape	Parameters
1			Input Layer	256×256×3	–	256×256×3	0
2			Patches	256×256×3	Input Layer	256×768	0
3			Patch Encoder	256×768	Patches	256×256	262400
4			Layer Normalization #1	256×256	Patch Encoder	256×256	512
5			Multi-Head Attention #1	256×256	Layer Normalization #1	256×256	3155200
6			Add #1	256×256 , 256×256	Multi-Head attention #1, Patch Encoder	256×256	0
7			Layer Normalization #2	256×256	Add #1	256×256	512
8	**Inception Module #1**		**Reshape #1**	256×256	**Layer Normalization #2**	16×16×256	0
			**Conv2D #1**	16×16×256	**Reshape #1**	16×16×96	24672
			**Conv2D #2**	16×16×256	**Reshape #1**	16×16×16	4112
			**Conv2D #3**	16×16×256	**Reshape #1**	16×16×64	**16448**
			**MaxPooling2D #1**	16×16×256	**Reshape #1**	16×16×256	**0**
			**Conv2D #4**	16×16×96	**Conv2D #1**	16×16×128	110720
			**Conv2D #5**	16×16×16	**Conv2D #2**	16×16×32	12832
			**Conv2D #6**	16×16×256	**MaxPooling2D #1**	16×16×32	8224
			**Concatenate #1**	16×16×64, 16×16×128 , 16×16×32 , 16×16×32	**Conv2D #3,** **Conv2D #4,** **Conv2D #5,** **Conv2D #6**	16×16×256	**0**
			**Reshape #2**	16×16×256	**Concatenate #1**	256×256	**0**
9			Add #2	256×256 , 256×256	Reshape #2, Add #1	256×256	0
10			Add #3	256×256 , 256×256	Add #2, Patch Encoder	256×256	0
11			Layer Normalization #3	256×256	Add #3	256×256	512
12			Multi-Head attention #2	256×256	Layer Normalization #3	256×256	3155200
13			Add #4	256×256 , 256×256	Multi-Head attention #2, Add #3	256×256	0
14			Layer Normalization #4	256×256	Add #4	256×256	512
15		**Inception Module #2**	**Reshape #3**	256×256	**Layer Normalization #4**	16×16×256	0
			**Conv2D #7**	16×16×256	**Reshape #3**	16×16×96	24672
			**Conv2D #8**	16×16×256	**Reshape #3**	16×16×16	4112
			**Conv2D #9**	16×16×256	**Reshape #3**	16×16×64	**16448**
			**MaxPooling2D #2**	16×16×256	**Reshape #3**	16×16×256	**0**
			**Conv2D #10**	16×16×96	**Conv2D #7**	16×16×128	110720
			**Conv2D #11**	16×16×16	**Conv2D #8**	16×16×32	12832
			**Conv2D #12**	16×16×256	**MaxPooling2D #2**	16×16×32	8224
			**Concatenate #2**	16×16×64, 16×16×128 , 16×16×32 , 16×16×32	**Conv2D #9,** **Conv2D #10,** **Conv2D #11,** **Conv2D #12**	16×16×256	**0**
			**Reshape #4**	16×16×256	**Concatenate #2**	256×256	**0**
16			Add #5	256×256 , 256×256	Reshape #4, Add #4	256×256	0
17			GlobalAveragePooling1D #1	256×256	Add #5	256	0
18			Dense #1	256	GlobalAveragePooling2D #1	64	16448
19			Dense #2 (Output Layer)	64	Dense #1	4 (for the Maize dataset), 38 (for the Plant-Village dataset)	262 , 2470
**Total Weight Parameters**						**6945574 (for the Maize dataset)** **6947782 (for the PlantVillage dataset)**	

The value written in bold font highlights the layers of Inception module in this table. These layers are the only difference between existing ViT model and our proposed TrIncNet model.

It can be perceived from **Tables 3**, **4** that the Inception module present in the TrIncNet model has used 32.67% fewer weight parameters compared to the MLP module present in the ViT model. This results in a 2.41% overall decrement in weight parameters from the ViT model to the TrIncNet model for both datasets. The results obtained during the experimentation of this research work are given in the next section.

## 5 Experimental results

The proposed TrIncNet network was trained and tested on two agricultural image datasets viz. PlantVillage and Maize dataset. The prediction performance of proposed network was evaluated on the validation and test subsets of both the datasets and comparative analysis was done with the existing ViT model and six state-of-the-art CNN architectures: VGG-19, GoogLeNet, ResNet-50, Xception, InceptionV3, and MobileNet.

### 5.1 Performance of the proposed network on Maize dataset

**Figure 5:**
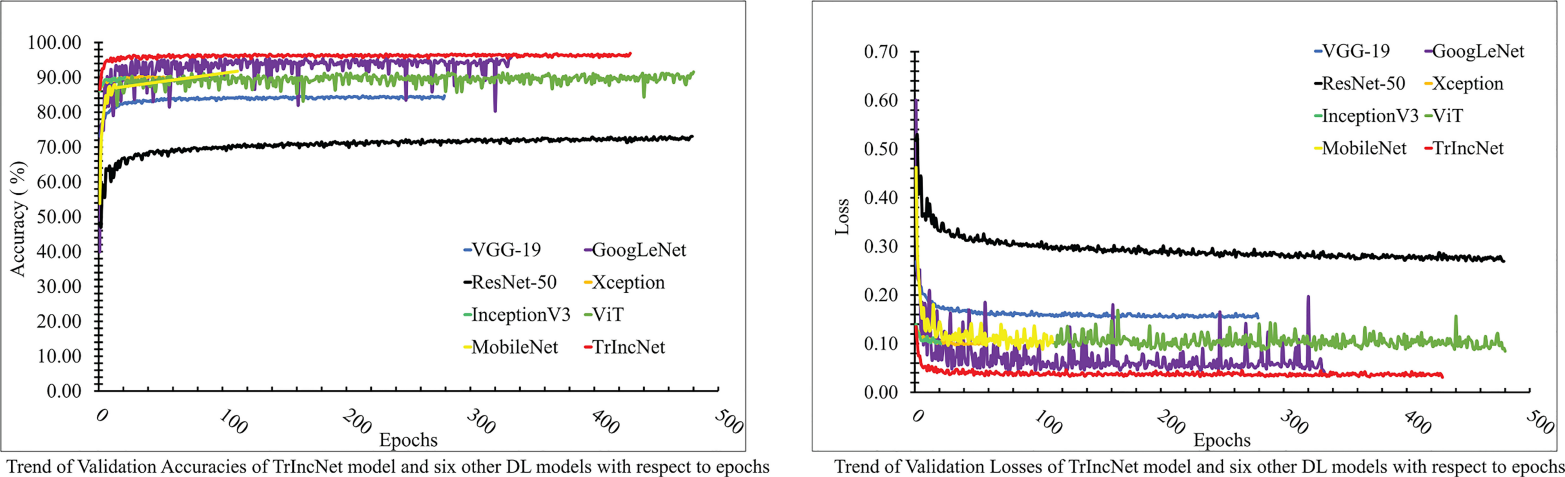
Plot of validation accuracies and validation losses of the TrIncNet model along with the ViT model and the six other state-of-the-art CNN architectures for the Maize dataset.

The plot of validation loss and validation accuracy of the TrIncNet model, along with the ViT model and six state-of-the-art CNN architectures for the Maize dataset, has been depicted in **Figure 5**. It can be observed from **Figure 5** that the proposed TrIncNet model attained the maximum validation accuracy, i.e., 97.0% and minimum validation loss, i.e., 0.035. Among the other six DL models, the GoogLeNet model attained second finest results for both validation accuracy and validation loss. Moreover, Xception and InceptionV3 models have achieved comparable accuracy, i.e., 90.38% and 90.23%, and comparable loss, i.e., 0.091 and 0.095. Other DL models which are used for comparison have attained validation accuracies in the range of 73.18% to 91.78% and validation losses in the range of 0.286 to 0.082.

**Figure 6:**
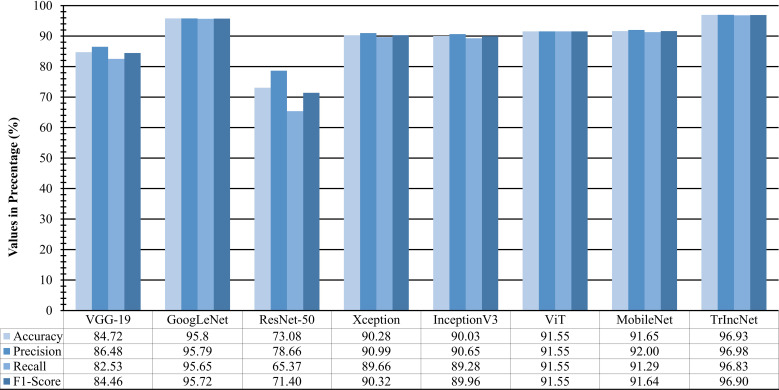
Comparison of f1-score, precision, recall, and accuracy attained by the proposed TrIncNet model along with the ViT model and six state-of-the-art CNN architectures for the Maize dataset.

To study the efficacy of the TrIncNet model more thoroughly, f1-score, precision, recall, and accuracy are also computed for the TrIncNet model along with the ViT model and six state-of-the-art CNN architectures on the Maize dataset’s test subset. These results have been given in **Figure 6**. It can be observed from **Figure 6** that the TrIncNet model achieved the best results for each above-mentioned metrics, i.e., 96.93% accuracy, 96.98% precision, 96.83% recall, and 96.9% f1-score on the Maize dataset. The Xception and InceptionV3 models have attained comparable results, and ResNet-50 have got minimum values for the aforementioned metrics. Moreover, GoogLeNet, ViT, MobileNet, and VGG-19 models achieved 95.72%, 91.55%, 91.64%, and 84.46% f1-score, respectively.

**Figure 7:**
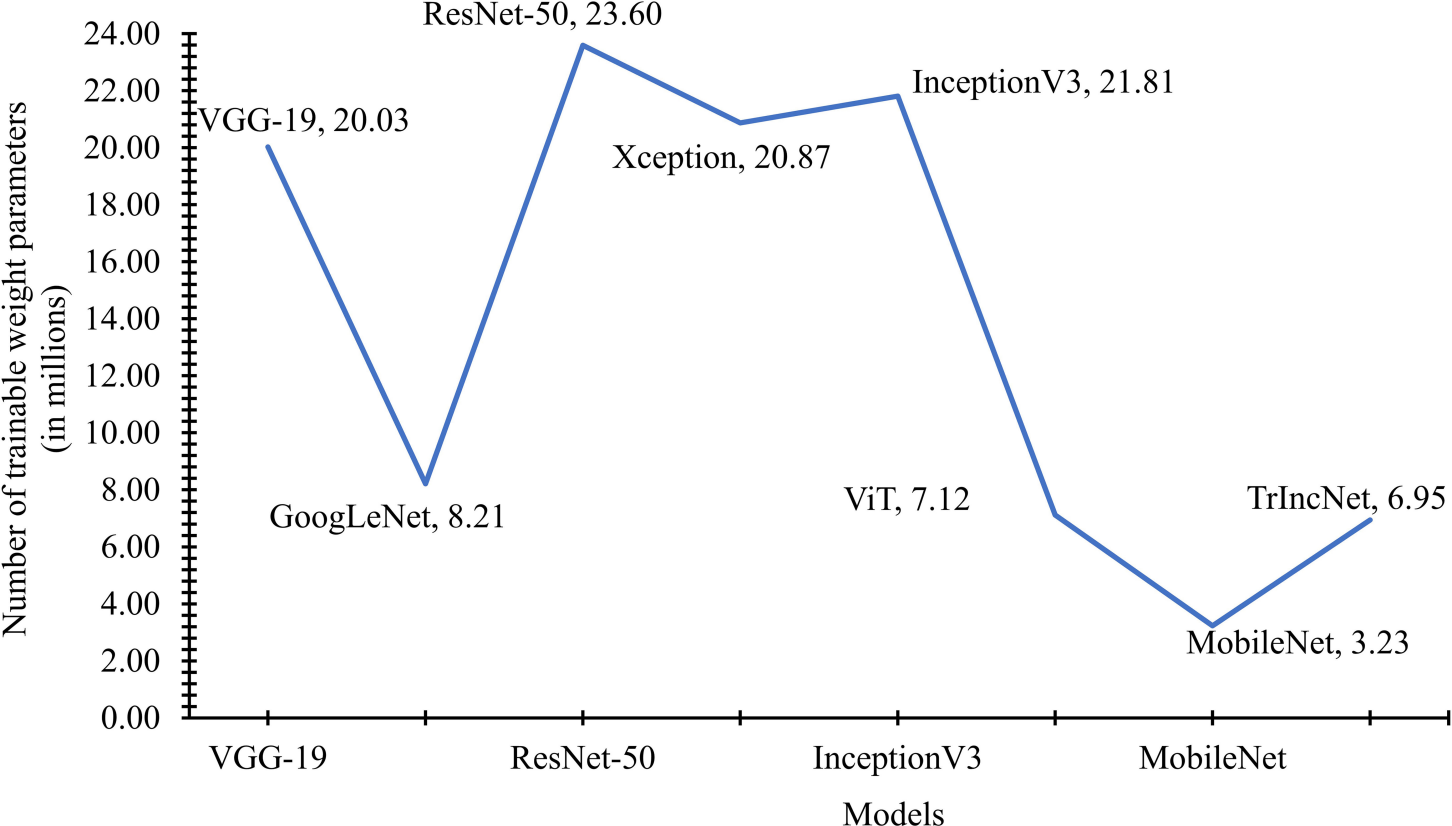
Comparison of the number of trainable weight parameters used by the TrIncNet along with the ViT model and six state-of-the-art CNN architectures trained on the Maize dataset.

The number of weight parameters utilized by the TrIncNet model, along with the ViT model and six state-of-the-art CNN architectures trained on the Maize dataset, have been compared in **Figure 7**. It is observed by seeing **Figure 7** that Xception, VGG-19, and InceptionV3 models require a comparable number of trainable parameters, i.e., 20.03 million, 20.87 million, and 21.81 million. Whereas ResNet-50 uses 23.60 million, and the GoogLeNet model uses 8.21 million trainable weight parameters. It can also be observed from **Figure 7** that the TrIncNet model requires 6.95 million trainable weight parameters, which is 2.41% lesser than the ViT model, which requires 7.12 million weight parameters. Although the MobileNet model have minimum trainable weight parameters, i.e., 3.23 million, but it did not perform well as compared to the proposed TrIncNet model.

The TrIncNet model’s performance on Maize dataset has been compared in **Table 5** with the research work done by Haque et al. (2022). The reason for comparing it with only this research work is that the TrIncNet model is trained on the same Maize dataset, which was used by Haque et al. (2022) in their research work.

**Table 5:** Comparison of the TrIncNet model’s performance with a recent research work present in the literature for the identification of Maize plant diseases.

Research Work	Techniques used	Dataset used	Type of dataset	Testing Accuracy	Number of trainable weight parameters (In millions)
(Haque et al., 2022)	InceptionV3 with Global Average Pooling layer	Maize dataset	Captured from field	95.99%	21.78
Proposed Work	TrIncNet model	Maize dataset	Captured from field	96.93%	6.95

It can be perceived from **Table 5** that the TrIncNet model achieved approximately one percent higher testing accuracy than the research work done by Haque et al. (2022) in detecting three diseases (MLB, TLB, and BLSB) of Maize plants under real-field conditions. Moreover, the TrIncNet model requires approximately 68.1% lesser trainable weight parameters than the research work done by Haque et al. (2022). In the next subsection, results obtained on the PlantVillage dataset are discussed.

### 5.2 Performance of the proposed network on PlantVillage dataset

**Figure 8:**
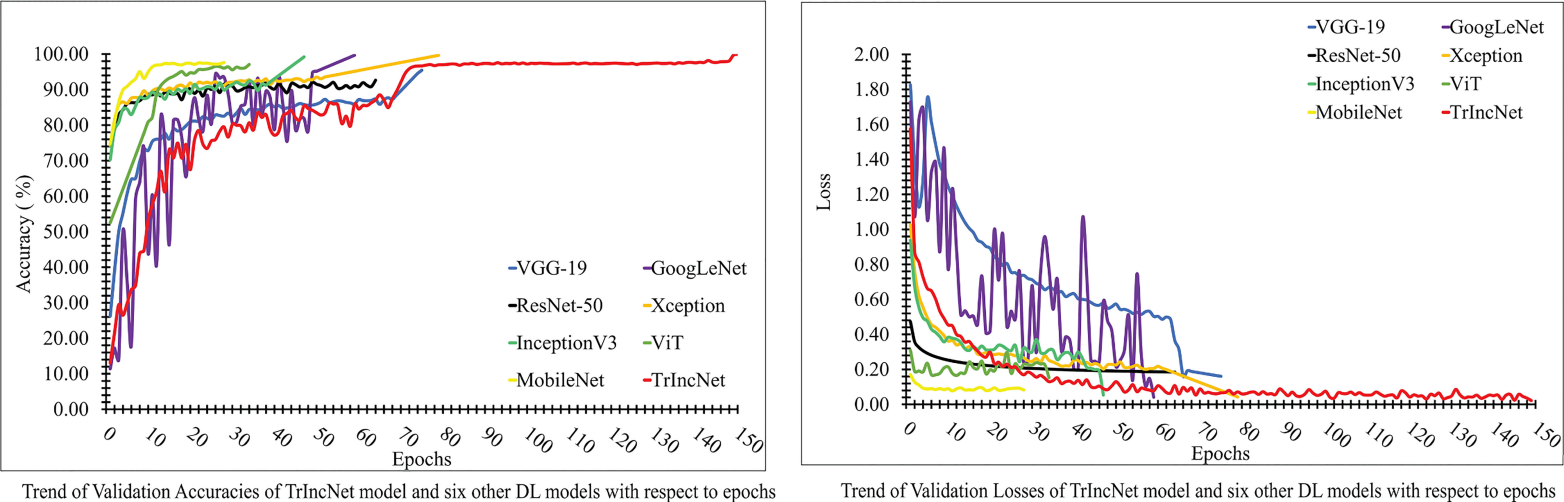
Plot of validation accuracies and validation losses of the TrIncNet model along with the ViT model and the six other state-of-the-art CNN architectures for the PlantVillage dataset.

In order to evaluate the TrIncNet model’s performance with the ViT model and six state-of-the-art CNN architectures, we have analyzed the trend of validation loss and validation accuracy w.r.t the epochs (depicted in **Figure 8**) obtained during the training. It has been observed by analyzing the plot given in **Figure 8**, that the Xception, GoogLeNet, and InceptionV3 models have attained comparable accuracies, i.e., 99.76%, 99.78%, 99.28%, and comparable loss, i.e., 0.04, 0.04, and 0.05. Furthermore, other DL models used for comparison have attained validation accuracies in the range of 92% to 97% and validation losses in the range of 0.18 to 0.08. It can also be observed from **Figure 8** that the proposed TrIncNet model attained highest validation accuracy and lowest validation loss, i.e., 99.95%, and 0.02, respectively.

**Figure 9:**
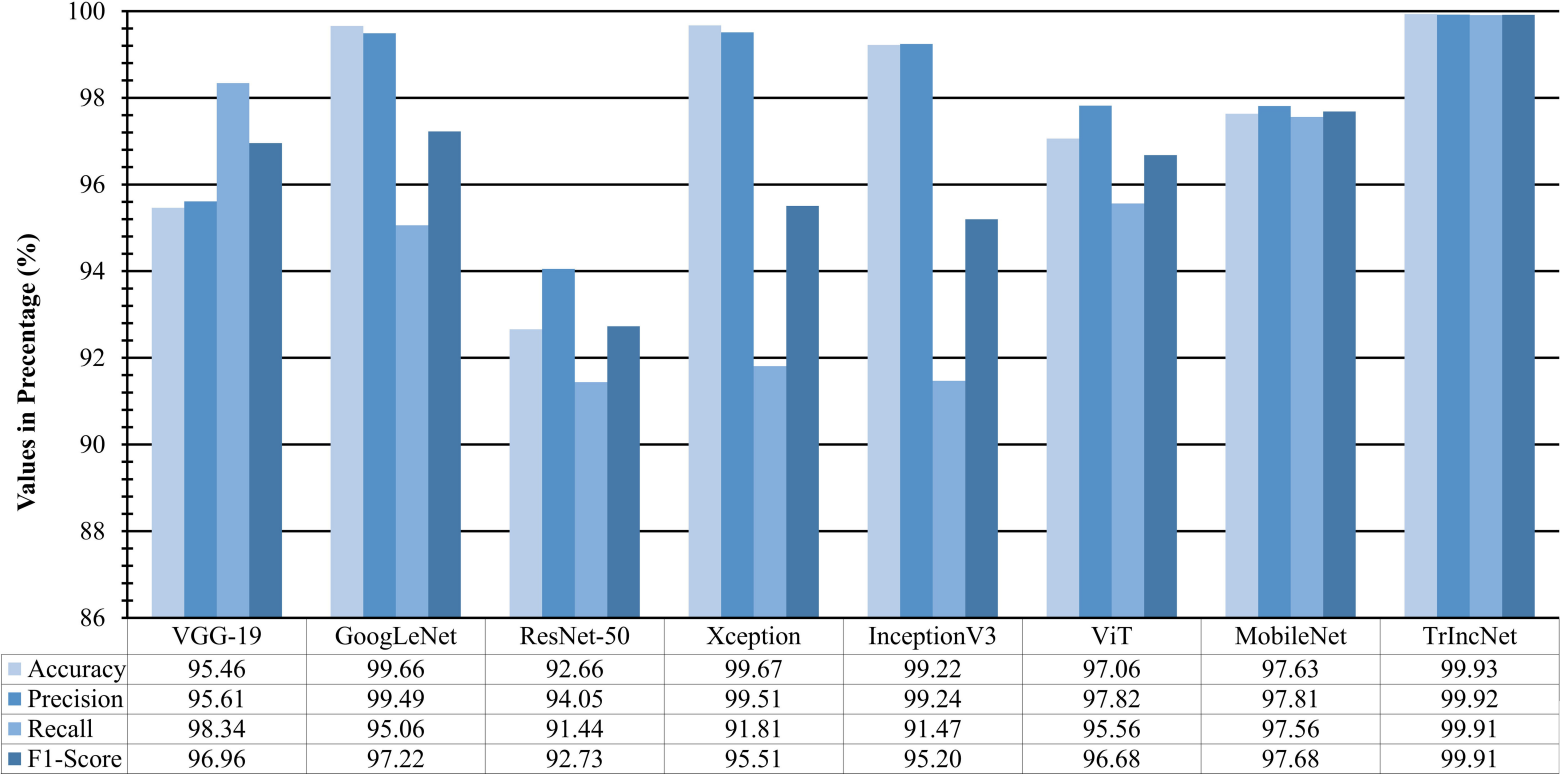
Comparison of f1-score, precision, recall, and accuracy attained by the proposed TrIncNet model along with the ViT model and six state-of-the-art CNN architectures for the PlantVillage dataset.

The performance of TrIncNet model along with ViT model and six state-of-the-art CNN architectures has been analyzed more thoroughly by computing f1-score, precision, recall, and accuracy on the test subset of PlantVillage dataset for all models. These results have been compared in **Figure 9**, and it can be observed from **Figure 9**, that the proposed TrIncNet outperformed the ViT model and six state-of-the-art CNN architectures with 99.93% accuracy, 99.92% precision, 99.91% recall, and 99.91% f1-score. Whereas, GoogLeNet, VGG-19, ViT, and MobileNet models achieved 97.22%, 96.96%, and 96.68%, and 97.68% f1-score, respectively.

**Figure 10:**
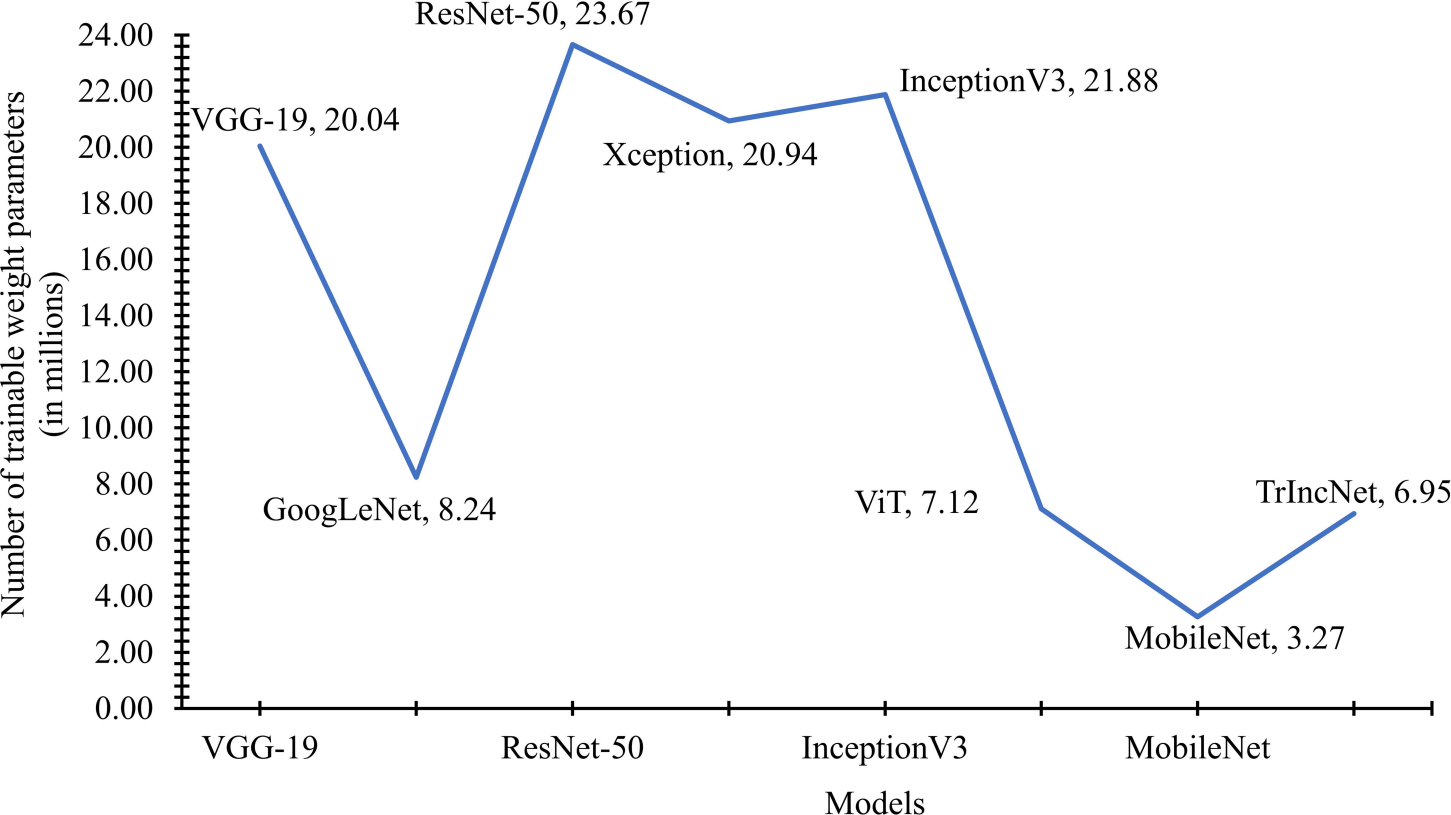
Comparison of the number of trainable weight parameters used by the TrIncNet along with the ViT model and six state-of-the-art CNN architectures trained on the PlantVillage dataset.

We have also calculated the number of weight parameters used by the TrIncNet model for the PlantVillage dataset and compared them with the weight parameters of the ViT model and six state-of-the-art CNN architectures on the same dataset. This comparison of weight parameters has been shown by a line chart in **Figure 10**. It can be seen by analysing the line chart given in **Figure 10** that the ResNet-50 and GoogLeNet models use 8.24 million and 23.67 million trainable parameters, respectively. Whereas the VGG-19, Xception, and InceptionV3 models require comparable weight parameters. Although, it can also be observed from **Figure 10** that the MobileNet model requires minimum trainable weight parameters, i.e., 3.27 million, but it did not perform well as compared to the proposed TrIncNet model.

The performance of the TrIncNet model on PlantVillage dataset has also been compared in **Table 6** with several recent research works present in the literature in which the PlantVillage dataset is used for model training. It can be observed from **Table 6** that the proposed model has attained state-of-the-art results by using a significantly lesser number of trainable weight parameters on the PlantVillage dataset as compared to other studies present in the literature.

**Table 6:** Comparison of the TrIncNet model’s performance with several recent studies present in the literature on PlantVillage dataset.

Research Work	Techniques used	Dataset used	Testing Accuracy	Number of trainable weight parameters (In millions)
Kaya and Gürsoy (2023)	Fused-DenseNet-121	PlantVillage dataset	98.17%	8.13
Ahmad et al. (2023)	DenseNet-169	PlantVillage dataset	99.5%	12.70
Atila et al. (2021)	EfficientNet-B5	PlantVillage dataset	99.91%	30.56
Proposed Work	TrIncNet model	PlantVillage dataset	99.93%	6.95

### 5.3 Ablation study

**Figure 11:**
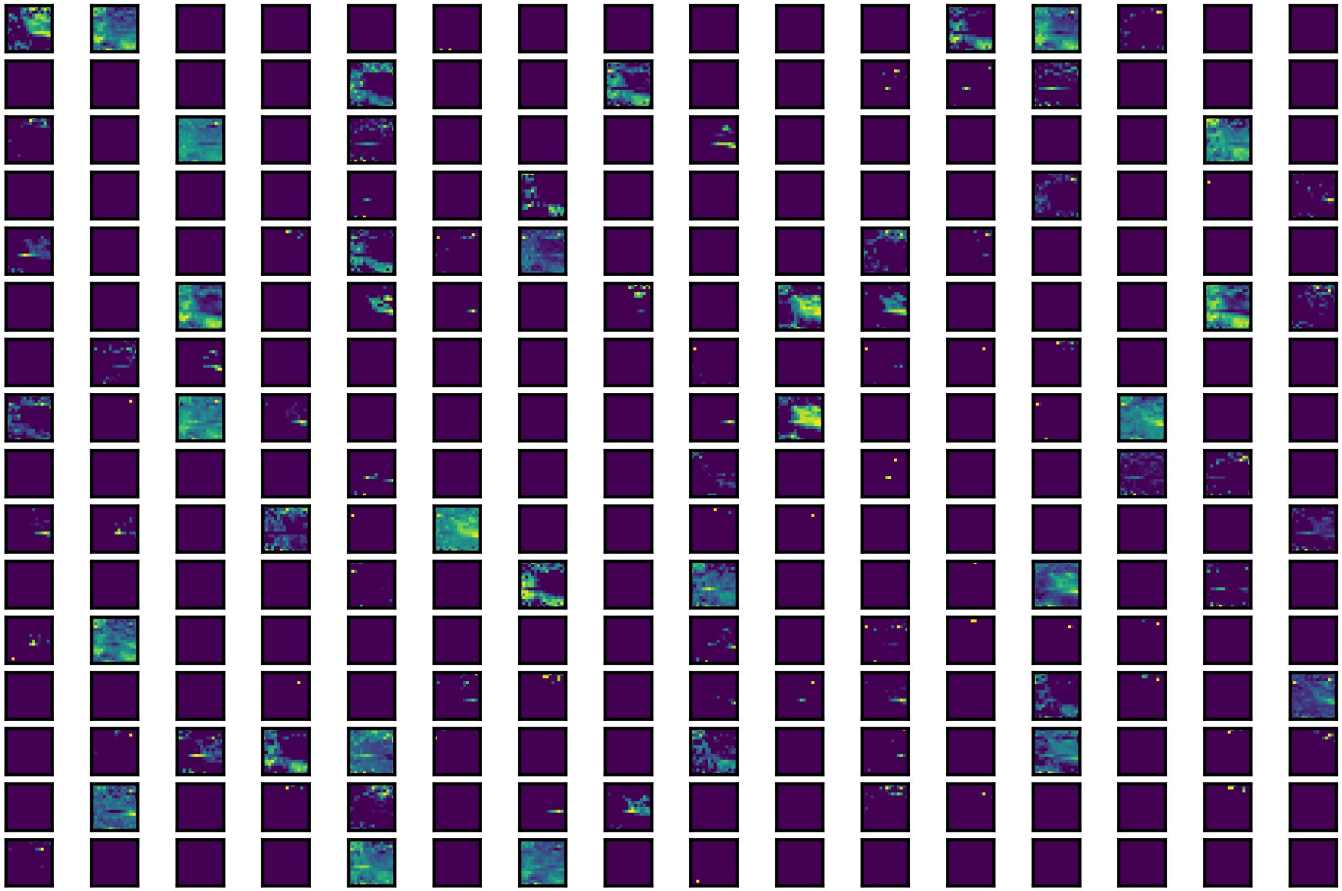
Visual representation of features extracted by MLP module present in the ViT model’s encoder block.

**Figure 12:**
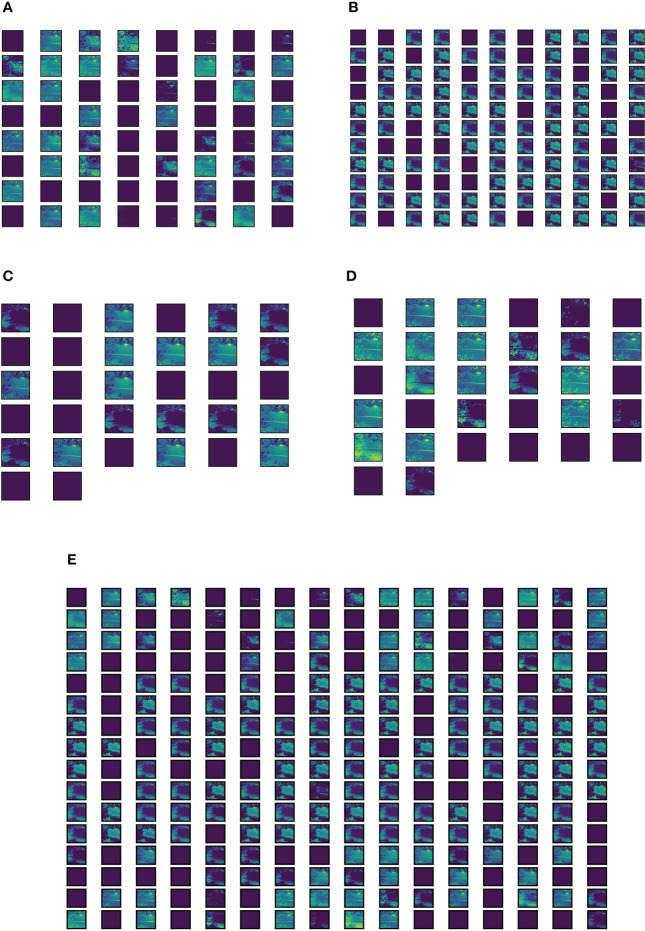
Visual representation of features extracted by the Inception module present in the Trans-Inception block. **(A)** Features extracted by 1 × 1 convolution operation. **(B)** Features extracted by 3 × 3 convolution operation. **(C)** Features extracted by 5 × 5 convolution operation. **(D)** Features extracted by 3 × 3 max-pooling operation. **(E)** Concatenation of all features extracted by 1 × 1, 3 × 3, 5 × 5 convolution operations and 3 × 3 max-pooling operation.

In order to visualize the feature extraction abilities of the MLP module of ViT model’s encoder block and the Inception module of Trans-Inception block, their extracted features are plotted in **Figures 11**, **12**, respectively. It can be seen from **Figure 11** that the MLP module present in ViT model’s encoder block is able to capture various features of leaf images. However, these features are very limited (as many feature maps shown in **Figure 11** are empty) and not very rich in quality because the MLP module is inefficient in capturing various spatial and temporal features of images.

The Inception module performs three convolution operations with 5×5 , 1×1 , and 3×3 filters and a 3×3 max-pooling operation simultaneously. The features extracted by individual operations of the Inception module are represented in **Figures 12A–D**, and the concatenation of all features extracted by all four operations of the Inception module is shown in **Figure 12E**. It can be observed from **Figure 12E** that the features captured by the Inception module are much richer in quality as compared to the MLP module. Moreover, the Inception module is able to capture more number of features than the MLP module. Hence, in this research work, the MLP module of ViT model’s encoder is replaced with the Inception module to form the Trans-Inception block, and these multiple Trans-Inception blocks are stacked together to form the novel TrIncNet model. In the next subsection, the TrIncNet model’s performance is compared with the ViT model and six state-of-the-art CNN architectures on the Maize dataset (which comprises real-field leaf images with complex backgrounds) and PlantVillage dataset, which acts as a benchmark dataset for plant disease detection problems.

It can be concluded from the above discussion that the TrIncNet model has achieved remarkable performance in diagnosing plant disease either in lab conditions or in real-field conditions with the minimum number of weight parameters. Therefore, it can be integrated with different IoT devices to assist farmers in identifying plant diseases at the earliest possible stage.

## 6 Discussion

This research aims to efficiently and effectively detect plant diseases by using digital images of their leaves and machine intelligence. The majority of the related studies have utilized various ML techniques (Khan et al., 2022), CNN-based techniques (Atila et al., 2021; Dhaka et al., 2021; Tiwari et al., 2021), ViT-based techniques (Thai et al., 2021; Wu et al., 2021; Borhani et al., 2022), or combination of ViT and CNN techniques (Zhang et al., 2022) to identify plant diseases, but all of these works have used the computationally expensive MLP module in the encoder block of ViT model. Thus, in the proposed TrIncNet model, the MLP module has been replaced with the Inception module in the ViT model’s encoder block to reduce the computational complexity of the ViT model. Furthermore, the TrIncNet model is much more resistant to the vanishing gradient problem than the ViT model, as it comprises of skip connections around each Trans-Inception block. Results obtained from the experimentation performed on two different datasets (Maize dataset and PlantVillage dataset) showed that despite of utilizing the minimum number of trainable weight parameters, the proposed TrIncNet model achieved the highest testing accuracy in identifying plant diseases *via* digital leaf images obtained either from labs or farmlands.

Experimental results revealed that the TrIncNet model attained higher testing accuracy than the ViT model. This trend of the results can be argued on the fact that in the Trans-Inception block of the proposed TrIncNet model, the MLP module is replaced with the Inception module, which can effectively and efficiently extract various spatial and temporal features from leaf images. This replacement also reduced the number of trainable weight parameters used by the proposed TrIncNet model, as the Inception module performs convolution and max-pooling operations which require lesser trainable weight parameters than the fully connected layers present in the MLP module. It can be seen from **Tables 5**, **6** that, on both datasets, the proposed model also got higher testing accuracy with a significantly lesser number of trainable weight parameters than the six state-of-the-art CNN architectures and the research work done by (Atila et al., 2021; Haque et al., 2022; Ahmad et al., 2023; Kaya and Gürsoy, 2023).

Conclusively, it can be said that the TrIncNet model proposed in this study has the potential to efficiently and effectively identify plant diseases *via* their digital leaf images captured either from the lab or agricultural fields with high accuracy. Moreover, the low computational complexity of the proposed model improves its training and inferencing speed. This study is also opened a new arena for further improvements in the ViT model’s architecture for plant disease detection and other image-based tasks. In this research work, the proposed model’s performance is evaluated on only two datasets; however, in the future, it is planned to train the model on other plant disease detection datasets that encompass leaf images with a wider range of diseases. Furthermore, the future work also includes the deployment of the proposed model on IoT devices such as UAVs, enabling real-time plant disease detection in agricultural fields.

## 7 Conclusion

Identifying plant diseases in their earliest possible infestation stage is one of the major research problems in the agricultural sector because it can potentially maximize crop yield and profit of the farmers. In order to solve this research problem, many researchers applied various ML techniques, CNN-based techniques, the combination of CNN and ML techniques, ViT-based techniques, or a combination of CNN and ViT for automatically diagnosing diseases in plants. However, none of the research works removed the MLP block from the ViT model’s encoder block, as it was a computationally expensive module as well as inefficient in extracting features from images. Hence, in this research work, a novel TrIncNet model was proposed, which contains multiple stacked Trans-Inception blocks. The proposed Trans-Inception block was designed by replacing the MLP module with the Inception module in the original ViT model’s encoder block. Moreover, in the TrIncNet model, each Trans-Inception block was surrounded by a skip connection which made the proposed model much more resistant to the vanishing gradient problem. The performance of TrIncNet model compared with the ViT model and six state-of-the-art CNN architectures (ResNet-50, VGG-19, GoogLeNet, Xception, InceptionV3, and MobileNet) using the PlantVillage dataset (benchmark dataset for plant disease detection problems) and Maize dataset (contains real-in-field leaf images with complex background). During experimental study, it was found that the TrIncNet model outperformed the ViT model and six CNN architectures with testing accuracies of 99.93% and 96.93% for the PlantVillage dataset and Maize dataset, respectively. Moreover, during experimentation, it was found that the proposed model used 6.94 million trainable weight parameters, which was the minimum among the ViT model and six state-of-the-art CNN architectures. The TrIncNet model’s performance has also been compared with other research works present in the literature, and its performance was found best among all of them.

## Data availability statement

The experimentation of this research work has been carried on two datasets: PlantVillage dataset and Maize dataset. The PlantVillage dataset, is openly available on https://github.com/spMohanty/PlantVillage-Dataset/tree/master/raw, and the Maize dataset will be available from the corresponding author on reasonable request.

## Author contributions

PG: Conceptualization, Methodology, Software, Writing - Original Draft, Visualization; PB: Project Administration, Writing - Review and Editing, Supervision, Formal analysis, Conceptualization, Methodology; SM: Writing Review and Editing, Formal Analysis, Resources; MH: Writing Review and Editing, Formal Analysis, Resources, Software; CD: Writing Review and Editing, Formal Analysis, Resources. All authors contributed to the article and approved the submitted version.

## Figures and Tables

**Figure A1:**
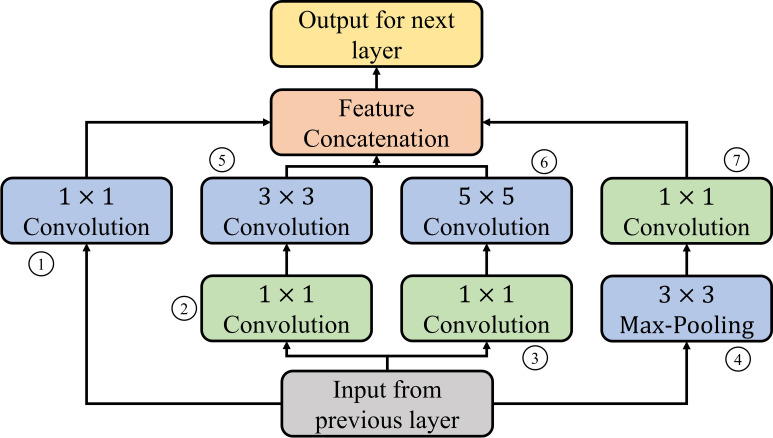
Block diagram of the Inception module.

## Appendix

**Asymptomatic analysis on the number of weight parameters used by the Inception module**

Let the input to the Inception module is $I\in {\mathbb{R}}^{M\times N}$ containing M patches, and each patch is of size N . Since the Inception module uses two-dimensional convolution operations, therefore I must be reshaped to $I^{\prime }\in {\mathbb{R}}^{\sqrt{N}\times \sqrt{N}\times M}$ , assuming that the value of N is a perfect square (i.e., $N\in 2^{2i},\;i=1,2,3,\ldots$ ). The asymptomatic analysis is done on each operation present in the Inception module (shown by numbers in **Figure A1**).

The number of weight parameters used in convolution operation can be expressed by equation (7) (Goodfellow et al., 2016).

(7)
$$W=I_{F}\times D\times k\times k$$

where:

W: number of weight parameters used in convolution operation

$I_{F}:$ number of filters applied

D: depth of input feature map

k: convolution filter’s size

Let the number of filters used in operation i is $F_{i}$ , where i=1,2,…, 7 (shown in **Figure A1**). Then the number of weight parameters used in each operation is computed in **Table A1**.

**Table A1:** Asymptomatic computation of weight parameters used by an inexpensive version of the Inception module.

Operation number	Input shape	Output shape	Number of weight parameters (asymptomatically)
**Operation 1**	$\sqrt{N}\times \sqrt{N}\times M$	$\sqrt{N}\times \sqrt{N}\times F_{1}$	$F_{1}\times M\times 1\times 1=F_{1}\times M=\;O\left(F_{1}M\right)$
**Operation 2**	$\sqrt{N}\times \sqrt{N}\times M$	$\sqrt{N}\times \sqrt{N}\times F_{2}$	$F_{2}\times M\times 1\times 1=F_{2}\times M=O\left(F_{2}M\right)$
**Operation 3**	$\sqrt{N}\times \sqrt{N}\times M$	$\sqrt{N}\times \sqrt{N}\times F_{3}$	$F_{3}\times M\times 1\times 1=F_{3}\times M=O\left(F_{3}M\right)$
**Operation 4**	$\sqrt{N}\times \sqrt{N}\times M$	$\sqrt{N}\times \sqrt{N}\times M$	0 (max-pooling operation does not require any weight parameters)
**Operation 5**	$\sqrt{N}\times \sqrt{N}\times F_{2}$	$\sqrt{N}\times \sqrt{N}\times F_{5}$	$F_{2}\times F_{5}\times 3\times 3=9\times F_{2}\times F_{5}=O\left(F_{2}F_{5}\right)$
**Operation 6**	$\sqrt{N}\times \sqrt{N}\times F_{3}$	$\sqrt{N}\times \sqrt{N}\times F_{6}$	$F_{3}\times F_{6}\times 5\times 5=25\times F_{3}\times F_{6}=O\left(F_{3}F_{6}\right)$
**Operation 7**	$\sqrt{N}\times \sqrt{N}\times M$	$\sqrt{N}\times \sqrt{N}\times F_{7}$	$F_{7}\times M\times 1\times 1=F_{7}\times M=O\left(F_{7}M\right)$

By combining the number of weight parameters used in each operation of the Inception module (given in **Table A1**), the total number of weight parameters used in this module can be calculated as $O\left(F_{1}M+F_{2}M+F_{3}M+F_{2}F_{5}+F_{3}F_{6}+F_{7}M\right)$ . If $F=\max\left(F_{1},F_{2},F_{3},F_{5},F_{6},F_{7}\right)$ , then the above expression can be simplified to $O\left(4\times FM+F^{2}\right)\Rightarrow O\left(FM+F^{2}\right).$ Hence, the total number of trainable weight parameters required to implement an inexpensive Inception module can be expressed by $W_{Inception}$ in equation (8).

(8)
$$W_{Inception}=\max\left(M^{2},F^{2}\right)=\{\begin{array}{c} O\left(M^{2}\right),\;F<M\\ O\left(F^{2}\right),\;F\geq M \end{array}$$
